# The Making of Doctors

**Published:** 1876-07

**Authors:** 


### The Making of Doctors.

Germany, with a population of 42,000,000, last year graduated six hundred and sixty physicians, rejecting one hundred and eighty applicants. In the same time the United States, with a population of 40,000,000, graduated three thousand physicians, and remorselessly turned them loose upon the community. We rather incline to the notion that in this country the supply is likely soon to exceed the demand.

